# A Gene-Oriented Haplotype Comparison Reveals Recently Selected Genomic Regions in Temperate and Tropical Maize Germplasm

**DOI:** 10.1371/journal.pone.0169806

**Published:** 2017-01-18

**Authors:** Cheng He, Junjie Fu, Jie Zhang, Yongxiang Li, Jun Zheng, Hongwei Zhang, Xiaohong Yang, Jianhua Wang, Guoying Wang

**Affiliations:** 1 College of Agriculture and Biotechnology, China Agricultural University, Beijing, China; 2 Institute of Crop Sciences, Chinese Academy of Agricultural Sciences, Beijing, China; Nanjing Agricultural University, CHINA

## Abstract

The extensive genetic variation present in maize (*Zea mays*) germplasm makes it possible to detect signatures of positive artificial selection that occurred during temperate and tropical maize improvement. Here we report an analysis of 532,815 polymorphisms from a maize association panel consisting of 368 diverse temperate and tropical inbred lines. We developed a gene-oriented approach adapting exonic polymorphisms to identify recently selected alleles by comparing haplotypes across the maize genome. This analysis revealed evidence of selection for more than 1100 genomic regions during recent improvement, and included regulatory genes and key genes with visible mutant phenotypes. We find that selected candidate target genes in temperate maize are enriched in biosynthetic processes, and further examination of these candidates highlights two cases, sucrose flux and oil storage, in which multiple genes in a common pathway can be cooperatively selected. Finally, based on available parallel gene expression data, we hypothesize that some genes were selected for regulatory variations, resulting in altered gene expression.

## Introduction

Following its domestication in southwestern Mexico ~9,000 years ago [1], maize has undergone further selection for local adaptation and through modern breeding, which has had significant effects on the genetic organization of maize [2]. Many studies have explored the dispersal of maize in both temperate and tropical regions [3], but few have examined genome-wide changes in the genetic constitution using genomic approaches [1,4,5]. Although genomic approaches can be applied to ancient DNA to explore evolution [6], most studies have used an alternative strategy—detecting evolutionary footprints by scanning the genomes with molecular markers in populations to reveal clues to past changes [7]. Under natural or artificial selection, the advantageous alleles and linked polymorphisms are found at a higher frequency, causing a reduction in genetic polymorphism, which has shaped adaptation and phenotypes.

Scans for selection in a population have largely been based on searches for distortions in the allele frequency spectrum or haplotypes under assumptions of neutrality [8]. The first genome-wide scans for selection, taking advantage of differentiation across populations, focused on average F_ST_ over multilocus windows [9], which had been applied to compare maize lines at various stages in their breeding history [10]. The cross population composite likelihood ratio (XP-CLR) test is another approach used to search for historical selection through population comparisons of the allele frequency spectrum [11,12]. Using this method, evidence for selection across the genome during maize domestication and improvement was evaluated in teosinte versus maize landraces, and landraces versus improved lines, respectively [4]. This same method was also used to reveal complex adaptation in maize worldwide by comparing tropical and temperate lines [13]. The XP-CLR approach is designed to detect “ancient” signature bottlenecks from several thousand years ago, while the cross population extended haplotype heterozygosity (XP-EHH) test, a haplotype-base approach, is designed to detect recently fixed or high frequency alleles in selective sweeps through population comparisons [14]. The XP-EHH test is insensitive to background selection and provides a less confounding approach for the systematic detection of positive selection in the genome [15].

Based on the genome-wide resequencing and analysis of wild, landrace, and improved maize lines, the genomic regions most affected by selection during domestication and improvement have been reported [4]. As an alternative to genomic resequencing, RNA sequencing technology, which defines both sequence and expression divergence [16], provides a cost effective way to explore even larger groups of inbred lines [13]. In addition, information about differential expression also provides an opportunity to examine the global consequences of gene expression in relation to selection pressure [17]. Here, we use 532,815 common Single Nucleotide Polymorphisms (SNPs) from our previous RNA sequencing study of 368 diverse maize lines to detect genomic regions affected by recent improvement by comparing diverse temperate and tropical inbred lines. The recent or ongoing positive selection of genomic regions was detected using a more powerful test based on Linkage disequilibrium (LD) and haplotypes [14], which will yield promising candidates for the molecular breeding of maize.

## Materials and Methods

### Plant materials and RNA sequencing

A collection of 368 maize inbred lines was studied for the detection of selective sweeps. The diverse group of maize lines, a subset of an association panel, were composed of 190 temperate lines and 178 tropical lines based on modeled genetic components (S1 Table), available pedigree information, and environmental adaptations[18]. This two-group classification included much more diverse temperate or tropical lines, which reduced false positives due to the founder effect of using fewer lines which will only decrease statistical power when conflict of grouping occurs. Sequencing RNA extracted from immature seeds 15 days after pollination (DAP) from the 368 inbred lines generated 25.8 billion reads. On average, 70.3% of the reads mapped to the B73 reference genome (AGPv2) were located in annotated genes (filtered gene set, release 5b60). For genes that had mapped reads at least 71.6% of the genes had >50% of the gene coding region covered by reads. In total, 1.02 million high-quality SNPs and 28,769 genes were detected. Because many of the lines used in this study are nearly homozygous, we do not expect >2 alleles at any given locus within an inbred line. The MaizeSNP50 BeadChip and the Sequenom MassArray iPLEX and PCR resequencing confirmed a subset of high-quality SNPs, which showed a concordance rate >96% [16]. For the purpose of selection scans, SNPs with minor allele frequency (MAF) of <5% were filtered out.

### Genome-wide scan for evidence of selection

Extended Haplotype Heterozygosity (EHH) is defined as the probability that two random samples are homozygous at all SNP loci. The Cross Population Extended Haplotype Heterozygosity (XP-EHH) test can detect alleles that have increased in frequency to the point of fixation or near-fixation in one of two populations based on their haplotype differences. XP-EHH has power over a very narrow time scale of positive selection. In our study, we performed a genome scan using the XP-EHH test between temperate and tropical maize lines. We estimated the genetic positions of the SNPs from a generalized additive model, which was fitted by a set of genetic markers with both known genetic positions and physical positions (http://ftp.maizegdb.org/MaizeGDB/FTP/B73_RefGen_v2_dumps/AGPv2_markers-7184.xlsx). Each gene region, including its 10-kb upstream and downstream regions, was taken as a unit to calculate the XP-EHH score of its SNPs. To estimate the genome-wide false discovery rate (FDR) in the selection scan, we divided the 368 maize lines into two ‘artificially identical’ populations and each pair of inbred lines in the two populations are nearly identical in their population genetic components (S2 Table). We calculated XP-EHH scores for all SNP markers in a population comparison using the same method as that used between temperate lines and tropical lines. At a given significance level, the FDR was calculated as P(*p*_*0*_ > *z*)/P(*p*_*1*_ > *z*), where *p*_*0*_ is the XP-EHH score from the permuted population, *p*_*1*_ is the XP-EHH score from the real population, *z* is the cutoff of XP-EHH scores at a given level from the real population, P(*p*_*0*_ > *z*) is the fraction of the number of XP-EHH scores >*z* in the permutated populations, and P(*p*_*1*_ > *z*) is the fraction in the real population.

### Identifying selected genomic regions

To find the genomic regions affected by selected alleles, we used a ‘Location and Extension’ method to identify selected regions with the empirical top SNPs. These SNPs were divided into two groups based on their potential selection in temperate or tropical maize lines. First, in each group, the gene with at least two SNPs in the top 5% of the XP-EHH scores is identified. Second, as an average distance of two adjacent genes containing SNP variations is approximately 100 kb, the region is iteratively extended by an up- or down-stream 100-kb region with less significant SNPs (in the top 10% of XP-EHH scores; S6 Fig). The flanking sigificant SNPs define the selected region. After location and extension, the intensities are represented by the highest scores in the extended regions. The regions including the top 1% SNPs were then identified as candidate selected regions, and the gene containing the SNP with the highest level XP-EHH score in each region was defined as the candidate gene under selection.

### Phylogenies analysis of candidate genes

Neighbor-joining trees were constructed from the candidate genes with more than 40 SNPs in the gene regions using MEGA7 (http://www.megasoftware.net/) with the parameters Test of Phylogeny = Bootstrap method (500), Model = p-distance, Gaps = Pairwise deletion. Trees were traversed using a custom R script to identify the common nodes of temperate or tropical lineages and the ratios of tropical lines co-segregating with temperate group as well as temperate lines co-segregating with tropical group were calculated. To avoid false positive, the candidate genes of which there were less than 50 lines in temperate or tropical group were excluded.

### Population genetic analyses

Population genetic summary statistics (*π*, *F*_ST_, Tajima’s *D*) were calculated for each gene coding region. We chose the SNPs located in the first transcripts of the maize filtered gene sets (B73 AGPv3, release 5b60) for analyses to reduce the confounders caused by different gene isoforms. Gene-based *π* and Tajima's *D* calculations were performed with VariScan (version 2.0.3), in which RunMode = 12 and the target region interval was set according to the transcript length. Gene-based *F*_ST_ was also used to measure the genetic differences between maize tropical and temperate subpopulations. Weir and Cockerham's estimator of *F*_ST_ was calculated by VCFtools (version 0.1.14) with the SNPs located in the first transcript of each gene. For whole-genome level calculations of *π* and *F*_ST_, all the RNA-seq SNP were included, with the same methods used as for the gene-based analyses.

### Annotation and enrichment analysis of maize genes

Gene annotation of protein kinases, transcription factors, and the ubiquitin-proteasome system followed the ProFITS [19] database for maize. Flowering time genes were collected from a study of ZmCCT [20], and the key maize genes were selected based on another study of maize gene evolution [21]. In order to assess the potential biological functions of the genes that underwent selection, the gene ontology (GO) enrichment was analyzed using the web toolkit agriGO [11]. When five or more mapped genes were grouped into each GO term, hypergeometric distributions were applied to test the significance against a background of the maize reference genome.

### Analysis of differential gene expression

To quantify gene expression, the RNA-seq reads were mapped to the maize genes (filtered-gene set, release 5b60), and reads per kilobases per million reads (RPKM) was calculated for each gene [22] as a representation of gene expression. Genes expressed in no less than 30 inbred lines in both temperate and tropical populations were used for differential expression analysis. The log_10_ of the average expression of a gene in each population was calculated, and a *t*-test was performed to identify whether this gene was differentially expressed at a significance level of 0.05.

## Results

### More selected genomic regions are found in temperate maize

To identify specific regions of the maize genome affected by selection during temperate or tropical maize improvement, we used a haplotype-base approach (XP-EHH test) to scan for selection signals based on population comparisons of the diverse maize inbred lines. The temperate lines retain 98.5% of the nucleotide diversity present in the group of maize inbred lines, while the tropical lines retain 97.0%. We applied a location-extension strategy to group the selection signals into the swept regions (S1 Fig). We focused our analyses on the regions that include the SNPs at the top 1% cutoff of XP-EHH scores.

**Fig 1:**
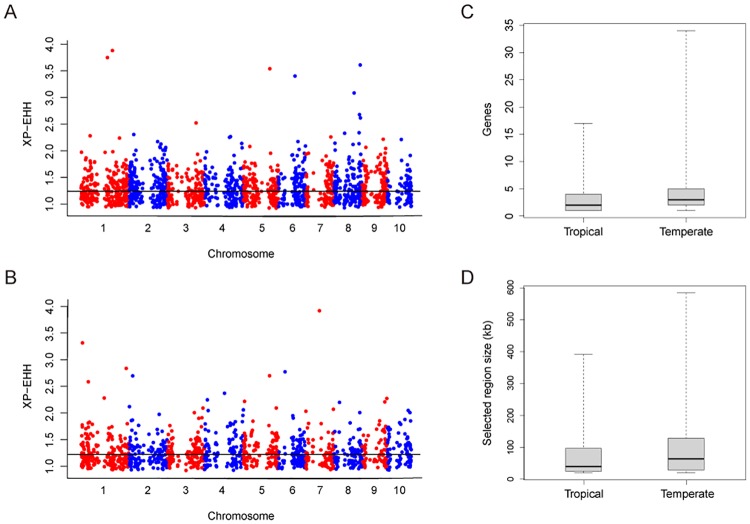
Genome-wide analysis of selected genomic regions in maize. Genome-wide XP-EHH scores for temperate (A) and tropical (B) selected regions. The cutoff lines represent an empirical cutoff for the top 1%. Distributions of region size (C) and gene counts (D) within selected regions in tropical and temperate maize lines.

In total, we identified 730 and 421 candidate selected regions that are specific to temperate maize and tropical maize, respectively, using an outlier approach (Fig 1A and 1B, S3 Table). We further estimated the genome-wide FDRs of these selected regions. The false discovery rate (FDR) for selected regions in temperate maize identified at the top 1% cutoff was 0.14, and for the tropical maize selected regions the FDR was 0.33. Compared with tropical selected regions, the temperate selected regions contain more genes and are larger. On average, selected regions in temperate maize contained 5.3 genes with a size of 95.2 kb covering 3.4% of the genome, while the selected regions in tropical maize contained 3.2 genes with a size of 71.1 kb covering only 1.5% of the genome (Table 1, Fig 1C and 1D). In addition, the values of Tajima’s *D*, a measure of deviation of the allele frequency distribution from that expected under the standard neutral model, were relatively lower in temperate maize than in tropical maize. The average value of Tajima’s *D* <-1 was -1.32 in temperate maize and -1.18 in tropical maize. These results indicate that temperate maize lines experienced more intensive selection than did the tropical lines, which is consistent with the fact that temperate maize has a longer history of modern intensive breeding than does tropical maize.

**Table 1:** Genomic regions selected in tropical and temperate maize lines.

	Region number^a^	Mean gene number^b^	Mean region size^c^	Coverage of genome^d^
**Temperate selected region**	730	5.29	95.2kb	3.38%
**Tropical selected region**	421	3.19	71.1kb	1.45%

^a^ Number of selected regions.

^b^ Average number of genes in the selected regions.

^c^ Average physical size of the selected regions.

^d^ The percentage of all selected regions in the maize genome.

### Regulatory genes are enriched in the selected regions

Previous studies have shown that genes of interest in crop domestication and improvement are likely to be regulatory genes [23]. In this study, the top five candidate genes under selection in temperate maize (*mybr84*, *tcptf31*, *bhlh173*, *CYCD1*, *ca5p2*) encode transcription factors (S3 Table). We also used three super classes of genes in regulatory and signaling pathways [19] to reveal functional overrepresentation of genes under artificial selection. Compared to all maize genes, only kinases were significantly enriched in the candidate temperate genes at a significance level of 0.01 (Table 2). We also found that both transcription factor genes and ubiquitin-related genes in the selected regions show significant enrichment in some specific families (S4 Table), such as bZIP, C3H, Ringfinger (hypergeometric-test, *P*-value <0.05). Our findings that regulatory genes are enriched under selection suggests that these genes have potentially important roles in characterizing temperate or tropical maize.

**Table 2:** The top 1% of genes in the selected genomic regions in temperate and tropical maize.

Super classes of genes	Background		Tropical candidate genes			Temperate candidate genes		
	number	%	number	%	*P*-value^d^	number	%	*P*-value^d^
PK^a^	1228	3.11%	21	4.76%	1.58E-02	40	4.48%	5.31E-03
TF^b^	2836	7.19%	42	9.52%	1.24E-02	67	7.51%	4.79E-02
UPS^c^	1205	3.06%	16	3.63%	8.06E-02	34	3.81%	3.12E-02

^a^ Protein Kinases.

^b^ Transcription Factors.

^c^ Ubiquitin-Proteasome System.

^d^ *P*-values of the hypergeometric-test.

### Gene expression is a target of recent maize selection

Gene expression is one of the most important features that reflects the function of a gene, and expression is a target of natural or artificial selection [24,25]. We used available gene expression data from immature ears as an indicator of relative expression across maize lines and compared differences in gene expression in selected genomic regions between temperate and tropical maize lines (Table 3). Candidate selected genes from both temperate and tropical maize showed a greater relative difference in expression than the genome background at a significance level of *P*-value <0.05. In particular, we found that nearly half of the candidate genes had significant changes in expression in selected genomic regions in temperate maize (S3 Table). Moreover, 20 temperate candidate genes and 13 tropical candidate genes showed a large and signifcant difference between the two groups (fold change ≥2, significant level of *P*-value <0.05), indicating that at least some genes in selected regions are under selection for regulatory alleles resulting in altered gene expression.

**Table 3:** Genes showing differences in expression in the maize genome and selected genomic regions.

	Filtered gene set		Tropical candidate genes			Temperate candidate genes		
	number	%	number	%	*P*-value^c^	number	%	*P*-value^c^
**Total**	32681		416			727		
**Significant**^a^	13529	41.4%	202	48.6%	3.01E-03	363	49.9%	3.01E-06
**Highly significant**^b^	9473	29.0%	146	35.1%	6.14E-03	262	36.0%	2.89E-05

^a^ Genes in which the expression differences between tropical and temperate populations are significant at *P*-values <0.05.

^b^ Genes in which the expression difference between tropical and temperate maize is significant at *P*-values <0.01.

^c^ *P*-values of the chi-square test.

### Artificial selection of key maize genes

Modern maize breeding has placed strong emphasis on the selection of existing variants, due to pressure to increase grain yield and intense phenotypic selection of inbred lines [3], which together have left their marks on the maize genome. To understand the influence of recent artificial selection, we focused on key maize genes that have been identified by visible mutant phenotypes [21]. We found that 13 candidate selected genes in temperate maize are key genes (S3 Table, Table 4), and the percentage of selected genes among key genes is significantly higher than the percentage of selected genes in a filtered gene set (hypergeometric-test, *P*-value < 0.05). This reflects the more intensive selection imposed on these functionally important genes in temperate maize. Functional analysis of 14 strongly selected key genes (the top 0.5%) in selected regions (Table 4) showed their potential roles in various biological processes, such as carbon metabolism (e.g., *sus1*, *incw1*), and kernel protein biosynthesis (*dzs18*; Table 4). Using gene expression data from RNA sequencing (see Methods), we also found that 9 of 14 strongly selected genes showed altered expression.

**Table 4:** Selected key genes in genomic regions selected during improvement in temperate maize.

Gene	Chromosome	Position	Annotation^a^	Selected region		Expression difference		
				Direction^b^	XP-EHH score^c^	TMP expression^d^	TPC expression^e^	*P*-value^f^
GRMZM2G442658	1	273983252–2739871476	alcohol dehydrogenase1, adh1	TPC	1.40767*	333.57	294.86	0.05
GRMZM2G087095	1	277310667–277348765	Zea mays MADS24, zmm24	TMP	-1.65527**	1.40	2.23	6.06E-6
GRMZM2G104546	2	172843794–172863857	aspartate kinase homoserine dehydrogenase2, akh2	TMP	-1.79858**	11.84	11.77	0.56
GRMZM2G174784	2	5514479–5518846	AP2-EREBP-transcription factor 197, ereb197	TPC	1.53975**	1.19	1.29	0.44
GRMZM2G026643	3	27551288–27558978	outer cell layer1, ocl1	TPC	1.66092**	4.01	3.47	4.29E-3
GRMZM2G109383	5	10855539–10861668	phosphoglucomutase2, pgm2	TMP	-1.29784*	130.18	136.06	0.08
GRMZM5G883855	5	16092084–16099014	ameiotic1, am1	TMP	-1.33957*	1.67	0.98	0.80
GRMZM2G087233	5	19437605–19439848	QM1 homolog1, qm1	TMP	-1.35767*	126.61	139.05	0.16
GRMZM2G139300	5	169454598–169459090	cell wall invertase1, incw1	TMP	-1.68728**	8.53	6.46	2.14E-3
GRMZM2G100018	6	121390345–121391179	delta zein structural18, dzs18	TMP	-1.4945**	4813.49	548.13	1.04E-7
GRMZM2G079440	6	137142553–137144384	dehydrin1, dhn1	TPC	1.57388**	10.79	8.74	0.07
GRMZM2G034647	8	120060780–120063628	cyclin1, cyc1	TMP	-1.49137**	1.64	1.63	0.48
GRMZM2G152908	9	122220190–122226863	sucrose synthase1, sus1	TMP	-1.44781**	267.39	251.33	8.69E-3
GRMZM2G067546	9	131161707–131169929	vacuolar sorting receptor homolog1, vacs1	TMP	-2.21364**	31.73	28.33	2.78E-3
GRMZM2G305046	9	139187765–139188857	histone2A1, his2a1	TMP	-1.86109**	145.66	117.16	0.04
GRMZM2G102161	9	146679028–146687322	Zea mays MADS8, zmm8	TPC	1.74751**	41.95	46.38	0.13
GRMZM2G171365	9	153812731–153832523	MADS1, mads1	TPC	2.26930**	16.48	26.62	2.86E-8
GRMZM2G147266	10	1279130–1283746	ferritin homolog2, fer2	TMP	-1.42697**	1.75	1.20	0.03
GRMZM5G803874	10	138485066–138487763	inhibitor of striate1, isr1	TMP	-1.64537**	1.08	1.09	0.25

^a^ Each candidate gene is annotated according to MaizeGDB (http://www.maizegdb.org).

^b^ Directions of selected regions. TMP means that this gene is in temperate selected region and TPC means in tropical selected region.

^c^ XP-EHH score is the most significant score of selected region.

^d^ The median gene expression in temperate population.

^e^ The median gene expression in tropical population.

^f^ The *P*-value of the *t*-test.

* selected key genes (top 1%).

** strongly selected key genes (top 0.5%).

#### Sucrose flux is strongly selected in temperate maize

**Fig 2:**
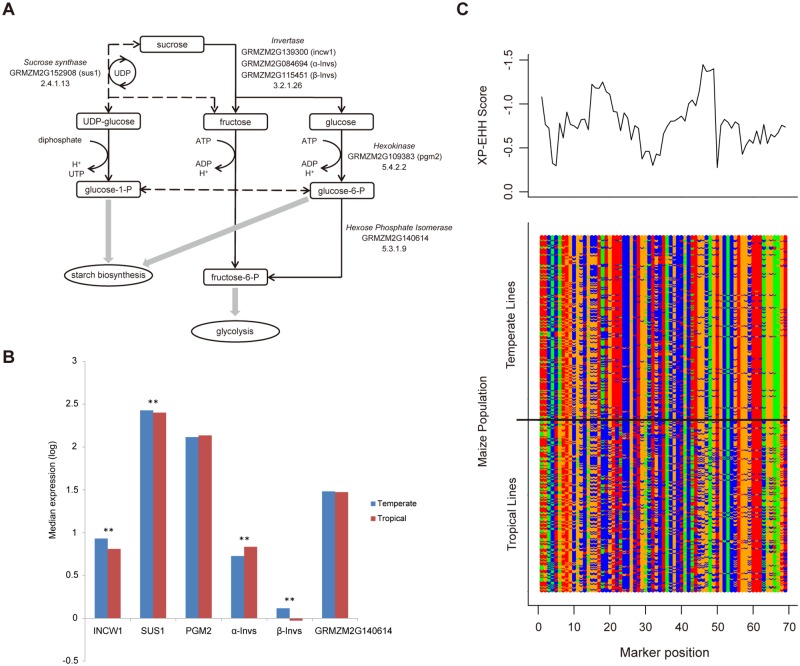
Selected genes in the sucrose catabolism pathway. (A) Diagram showing the sucrose catabolism pathway with six genes selected during maize improvement. (B) Expression levels of six selected sucrose catabolism genes in tropical and temperate maize lines. The double stars represent highly significant expression differences between tropical and temperate lines. (C) XP-EHH scores and haplotypes of SNPs in one selected gene (GRMZM152908; *sus1*).

Using gene ontology (GO) enrichment analysis, the selected genes in temperate maize were predicted to be involved in various biosynthetic processes (S2 Fig). Sucrose catabolism, a primary biosynthetic process (Fig 2A), plays a central role in carbon partitioning and biomass accumulation, and can only be catalyzed by two types of enzymes, sucrose synthase (SUS) and invertase (INV). An acid invertase gene (*incw1*) and the *sus1* gene were strongly selected only in temperate maize (Fig 2C, Table 4), suggesting that regulation of the sucrose pathway is the target of temperate selection. This is further supported by the fact that two genes, GRMZM2G109383 (*pgm2*) and GRMZM2G140614 (Fig 2A), encoding enzymes that act directly downstream, hexokinase (HXK) and hexose phosphate isomerase (HPI), were also under strong selection in temperate maize (S5 Table). In addition, we found two strongly selected neutral/alkaline invertase genes (top 0.5%), GRMZM2G084694 and GRMZM2G115451, which are orthologous to AT1G5650 (*α-A/N-Inv*) and AT1G72000 (*β-A/N-Inv*) in *Arabidopsis*, and differ in structure from the acid invertases [26]. These three selected invertases likely function in different subcellular locations, suggesting a precise coordination of cellular sucrose flux during temperate maize improvement. Directed changes in gene expression in these invertases in temperate maize suggests selection of their *cis*-acting regulatory regions (Fig 2B).

#### Oil storage controlled by selection of multiple genes

**Fig 3:**
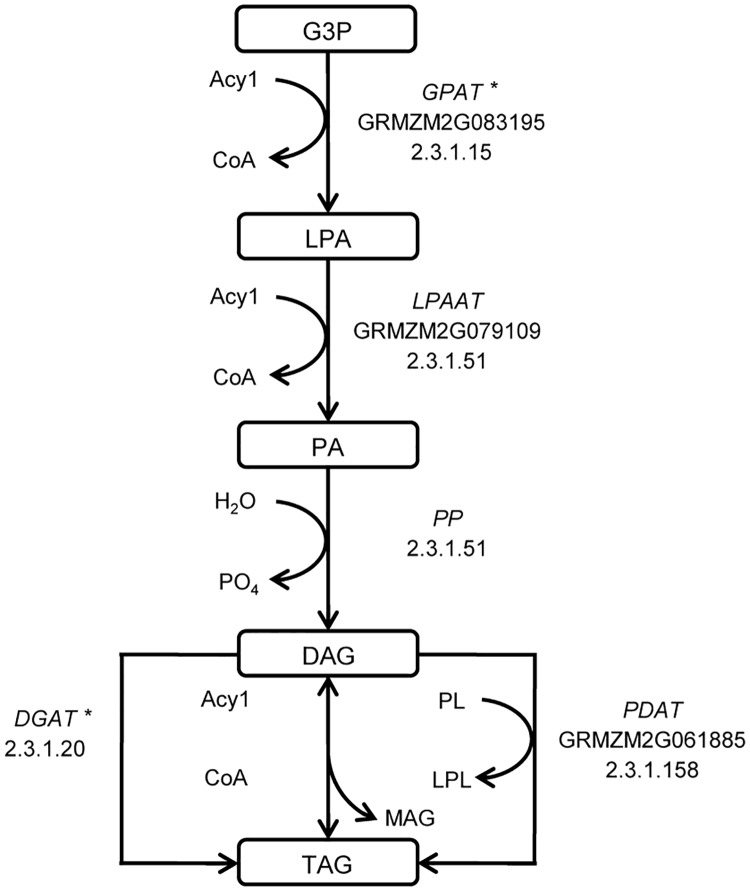
Selected genes in the triacylglycerol (TAG) biosynthesis pathway. Three genes (GRMZM2G083195, GRMZM2G079109, and GRMZM2G061885) that encode enzymes in the TAG biosynthesis pathway (GPAT, LPAAT, and PDAT) were strongly selected in temperate maize. The asterisks indicate candidate genes identified in a previous GWAS study of maize oil [27].

Fatty acids, as a highly reduced form of carbon, are one of the primary storage compounds in maize kernels, providing nutrients for subsequent germination and early development. Triacylglycerols (TAGs) are a major storage lipid (Fig 3, Table 5). Three genes (GRMZM2G083195, GRMZM2G079109 and GRMZM2G061885) that encode the enzymes in the TAG biosynthesis pathway (GPAT, LPAAT, and PDAT), were strongly selected in temperate maize lines, suggesting precise control of oil storage. Among these genes, *GPAT* showed an association with lipid content. *DGAT1-2* is one of the most significant loci associated with oil content [27], and it drives the final acylation to generate TAGs, depending on acyl CoA; *DGAT1-2* was not detected as a selected gene in temperate maize. However, a *PDAT* gene, which belongs to an acyl CoA-independent pathway for the production of TAGs, was found to be under strong selection. Moreover, two additional genes (GRMZM2G152105 and GRMZM2G003501) encoding fatty acid elongation enzymes were also under selection in temperate maize. Promisingly, an orthologue (GRMZM2G167576) of *LEC1*, which regulates the enzyme-coding genes of fatty acid biosynthesis at the transcriptional level [28] is strongly selected (top 0.5%). This implies that not only the enzyme-coding gene regions but also the regulatory gene regions were under selection to affect oil storage.

**Table 5:** Candidate selected genes involved in oil biosynthesis in selected genomic regions in temperate maize.

Gene	Chromosome	Position	Annotation^a^	Selected region	
				Level^b^	XP-EHH score^c^
GRMZM2G003720	2	48997817–49000991	acyl-CoA thioesterase, *ACT*	0.01	-1.41008
GRMZM2G061885^d^	2	184692047–184698536	phospholipid:diacylglycerol acyltransferase, *PDAT*	0.005	-1.46748
GRMZM2G079109	2	194036492–194041722	1-acylglycerol-3-phosphate O-acyltransferase, *LPAAT*	0.005	-1.68848
GRMZM2G083195	3	178181704–178185282	glycerol-3-phosphate O-acyltransferase, *GPAT*	0.005	-1.63522
GRMZM2G003501	4	166828995–166830916	very-long-chain 3-ketoacyl-CoA synthase	0.005	-1.51649
GRMZM2G167576	9	36849526–36850583	*cadtfr12*, orthology to LCE1 in *Arabidopsis*	0.005	-1.56933
GRMZM2G152105	9	122514778–122520703	β-ketoacyl-acyl-carrier-protein synthase I	0.01	-1.40285

^a^ Candidate genes were annotated according to MaizeGDB (http://www.maizegdb.org)

^b^ Significance levels for genes in all selected candidate regions

^c^ XP-EHH score is the most significant score of the selected region

^d^ This gene is a candidate gene identified in a previous GWAS study [27]

*Some flowering genes were newly selected during recent improvement*. Previous research suggests that improvement and adaptation in maize may not have been a sequential and discrete process, but was rather an overlapping process [13]. Thus, we focused on the selected genes involved in flowering, one of most important gene sets for adaptation, by examining two hundred flowering candidate genes from a previous study [20]. We detected six candidate selected genes and 24 swept genes in the selected regions that may be involved in flowering (S3 Table). Most of these genes are located on chromosomes 2, 5, and 8 and overlapped with two known flowering QTLs [20] (S3 Fig). We identified two candidate selected maize genes with homology to genes related to the *CO/FT* module (S4 Fig). Both of these genes, one homologous to the *CO* repressor *COL9* and the other homologous to the *FT* repressor *TOE1*, were found in the genomic regions under selection in tropical maize. These findings indicate that the regulators of the *CO/FT* module were likely to be selected in maize for improvement of yield and yield stability through fine-tuning of the flowering process.

## Discussion

Using gene-oriented haplotype comparisons, and taking advantage of the differentiation between tropical and temperate maize germplasm, we performed comprehensive analyses of alleles that underwent selection during recent improvement or modern breeding after the geographical dispersal of maize using SNPs detected in RNA sequencing. Our main finding of interest was that the selected candidate genes in temperate maize were enriched for biosynthetic processes, which indicated that recent intensive improvement efforts targeted downstream genes to modify trait performance without pleiotropic effects. Analysis of these candidate genes highlights three cases where multiple genes in a common pathway can be cooperatively selected, which revealed the traits under improvement. This is consistent with the coordinated consequences of natural selection in humans [14]. With available gene expression data, we can hypothesize that artificial positive selection pressure partly resulted in altered expression among the selected genes.

There are two modern approaches to detect the characteristic signals of positive selection; site frequency spectrum (SFS), and extended haplotype heterozygosity (EHH). Because EHH signatures can detect positive selection in fewer than 1200 generations [29], the EHH-based method should have more power to detect the signals of selective sweeps from recent artificial selection and maize breeding. Here, we chose a cross population EHH (XP-EHH) method, taking advantage of population comparisons between temperate and tropical maize populations. Considering that the SNP set we used originated from transcribed regions of the genome, we subsequently designed a gene-oriented approach to locate and extend the genomic regions that had experienced a selective sweep, which should be applicable to other genome-wide selection studies using cost-effective RNA sequencing. It is important to note that genomic regions identified using “outlier” approaches are not all expected to be targets of selection. SNPs in the tails of the empirical distribution can be false positives caused by historical demographic events [14]. We made every attempt to avoid these false positives by using data from much more diverse lines that are derived from multiple founders (S1 Table) and assessed the FDR of the detected signatures. To further identify stronger candidate genes, we also calculated the ratio of temperate or tropical lines that co-segregated with the incorrect group by constructing phylogenetic trees of these genes. Finally, we acquired the co-segregation ratios of 324 temperate and 100 tropical candidate genes and categorized them into different classes (S6 Table). Among them, 141 temperate candidate genes and 15 tropical candidate genes showed low co-segregation ratios less than 0.20 (S3 Table). Combing with the XP-EHH scores, a candidate gene that has a high XP-EHH score as well as a low co-segregation ratio should be a stronger and more reliable selected one in our study.

A previous report on genome-wide selection scans aimed to identify loci with an overall reduction of diversity for domestication or improvement [4]. Instead, it was the objective of our study to identify temperate- or tropical-specific selection, and the loci with an overall diversity reduction should be excluded. Only a small percentage, 8.1% and 7.1%, respectively, of temperate and tropical selected genomic regions overlapped with the aforementioned regions that were selected for improvement, indicating that at least most of the loci detected in our study are specific for selection in either temperate or tropical maize. Compared with all selected regions detected, the overlapping regions showed a larger range (S5 Fig). Among the overlapping regions, 35 have exactly the same candidate genes. These regions showed a diversity reduction in all 35 temperate or tropical lines included in a previous report [4], but had a high level of diversity in the tropical maize lines used in our study. These cases may be due to a founder effect from using a smaller set of lines and a relatively short time span of improvement, during which mutation has not acted to produce new variations. Comparing with another study in 2016 [12], 5 temperate selected regions were found overlapping with the domestication regions identified in their study (S3 Table), indicating a number of domestication loci could be under further selection in maize improvement.

A recent published study on differentiation between tropical and temperate maize with emphasis on adaptation, used XP-CLR to detect “ancient” signals during adaptation of maize [13], while our study focused on recent maize improvement that changed the genome constitution using XP-EHH. Only 42 of 730 temperate and 21 of 421 tropical selected regions identified by our XP-EHH test overlapped with results of the Liu et al. study, suggesting that there were significant differences between the selection signatures detected by these two studies. This is consistent with the fact that outlier-based genome scans utilizing either SFS or haplotype approaches to identify genomic regions as targets of positive selection had inconsistent overlap [30].

Only a small number of the newly-identified candidate genes have been studied with regards to their function in maize; one example is the temperate gene candidate *sus1* that is known to affect carbon partitioning and biomass accumulation [31]. Nucleotide diversity in the *sus1* genic region is lower in temperate lines (*π* = 0.00477) than in tropical lines (*π* = 0.00737). A fraction of candidate genes have stronger selection signals than these previously-identified genes that underly morphological and physiological changes. For example, the temperate candidate gene GRMZM2G167576, an orthologue of *LEC1*, shows strong evidence of positive selection (XP-EHH score = -1.569) and the low Tajima’s *D* value (Tajima’s *D* = -0.437) also indicates the selection. Because ~10% of the selected regions overlap with association signals of kernel weight traits (data not shown), these candidate genes, together with the swept genes, should prove useful in dissecting existing quantitative trait loci (QTLs).

The majority of maize production takes place in temperate regions of the world. However, tropical maize lines include stress related genes, that are absent in temperate maize. The selected genes identified in tropical maize, which reflect their importance for adaptation to tropical environments, provide a valuable gene pool for temperate maize improvement in the face of ongoing climate change. Tropical maize lines generally are more drought tolerant than temperate maize lines [32]. The drought-related genes *dhn1* [33] and *olc1* [34] have been strongly selected in tropical maize, suggesting potential roles for their selected alleles in drought tolerance in tropical maize. These selected alleles collectively constitute the genetic basis of drought tolerance in tropical maize.

The genomic landscape opens up new avenues for exploration of global DNA divergence in response to selection. RNA sequencing, a cost-effective sequencing technology, can detect sequence variation in transcribed regions and provides additional gene expression data in the studied lines. This can be integrated into the study of gene performance and differentiation under adaptation and selection during breeding. In combination with novel approaches to QTL mapping, these genomic strategies will facilitate the development of new cultivars in the face of a changing climate.

## Supporting Information

S1 Fig Computational pipeline used to identify selected regions of the maize genome in this study. (PDF)

S2 Fig GO analysis of candidate genes in the top 5% of selected genomic regions in temperate maize lines. (A) GO analysis of candidate genes in temperate selected regions. (B) Enrichment analysis of GO annotations of candidate genes in selected genomic regions in temperate lines.
(PDF)

S3 Fig Selected candidate and selective sweep flowering-time genes in maize genome. Distribution of flowering-time genes on the ten maize chromosomes. Vertical black hash marks at the bottom of each box represent the 730 and 421 selected genomic regions identified in temperate and tropical maize lines on the chromosomes based on their physical location on the maize AGPv2 reference genome. Green boxes and the letter “c” on the physical maps represent centromeres. Blue dots represent flowering-time genes in temperate selected regions and red dots represent flowering-time genes in tropical selected regions. The chromosomal positions and lengths of flowering-time QTLs are indicated by the orange boxes.
(PDF)

S4 Fig Selected candidate genes in the *CO/FT* module. Genes shown in red were selected in tropical maize lines.
(PDF)

S5 Fig Size distribution of selected genomic regions identified in this study and that of Hufford et al [4]. (PDF)

S6 Fig Graphical representation of the method used to identify and extend selected genomic regions. (PDF)

S1 Table Temperate and tropical maize inbred lines. ^a^SS, Stiff Stalk. ^b^NSS, Non-Stiff Stalk. ^c^TST, Tropic and Subtropic. The population structure was estimated according to the methods in Fu et al. 2013 [16].
(XLSX)

S2 Table Two ‘artificially identical’ populations for false discovery rate (FDR) estimation. ^a^SS, Stiff Stalk. ^b^NSS, Non-Stiff Stalk. ^c^TST, Tropic and Subtropic
(XLSX)

S3 Table Selected genomic regions in temperate and tropical maize lines. (XLSX)

S4 Table Significant enrichment of candidate selected genes in transcription factor families and ubiquitin pathways. ^a^The number of functional genes in the whole maize genome. ^b^*P*-value of the hypergeometric-test.
(DOC)

S5 Table Genes located in selected genomic regions. (XLSX)

S6 Table Summary of co-segregatuion ratios for candidate genes. (XLSX)
