# Supplementary figures and images for: A Gene-Oriented Haplotype Comparison Reveals Recently Selected Genomic Regions in Temperate and Tropical Maize Germplasm
Source: PLoS One. 2017 Jan 18;12(1):e0169806. doi: 10.1371/journal.pone.0169806 (PMC5242465; doi:10.1371/journal.pone.0169806)

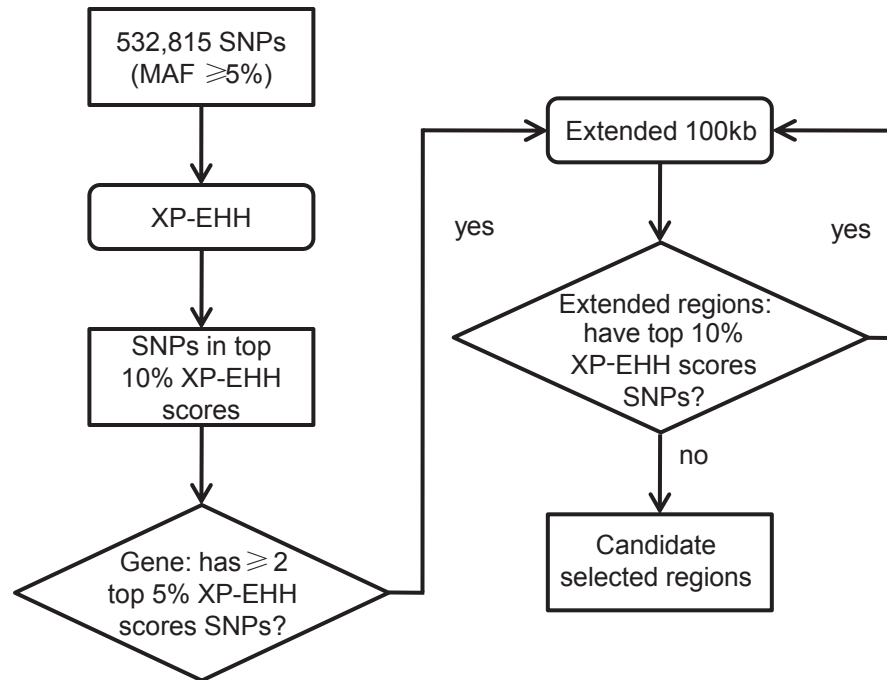

Supplement: S1 Fig — (PDF) [file pone.0169806.s001.pdf]

A

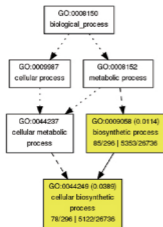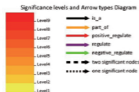

B

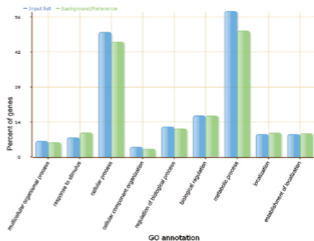

Supplement: S2 Fig — (A) GO analysis of candidate genes in temperate selected regions. (B) Enrichment analysis of GO annotations of candidate genes in selected genomic regions in temperate lines. (PDF) [file pone.0169806.s002.pdf]

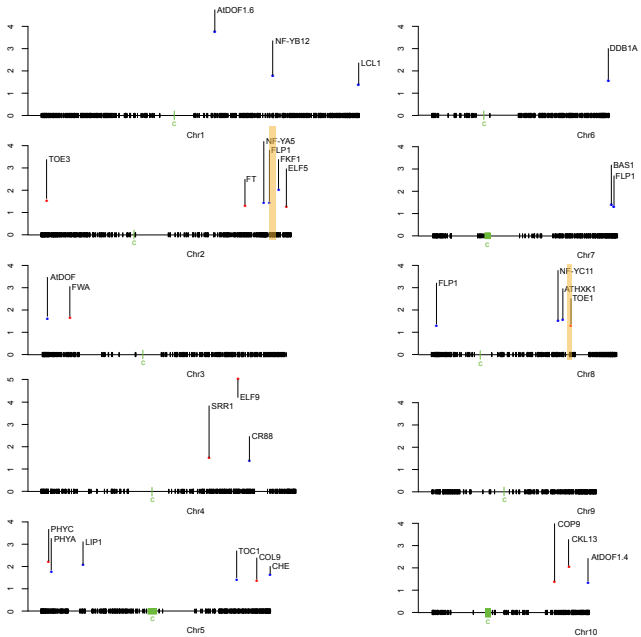

Supplement: S3 Fig — Distribution of flowering-time genes on the ten maize chromosomes. Vertical black hash marks at the bottom of each box represent the 730 and 421 selected genomic regions identified in temperate and tropical maize lines on the chromosomes based on their physical location on the maize AGPv2 reference genome. Green boxes and the letter “c” on the physical maps represent centromeres. Blue dots represent flowering-time genes in temperate selected regions and red dots represent flowering-time genes in tropical selected regions. The chromosomal positions and lengths of flowering-time QTLs are indicated by the orange boxes. (PDF) [file pone.0169806.s003.pdf]

GRMZM2G075562

COL9

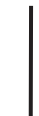

CO

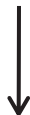

FT

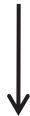

Flowering

miR172

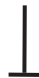

TOE1

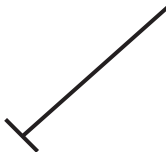

GRMZM2G700665

Supplement: S4 Fig — Genes shown in red were selected in tropical maize lines. (PDF) [file pone.0169806.s004.pdf]

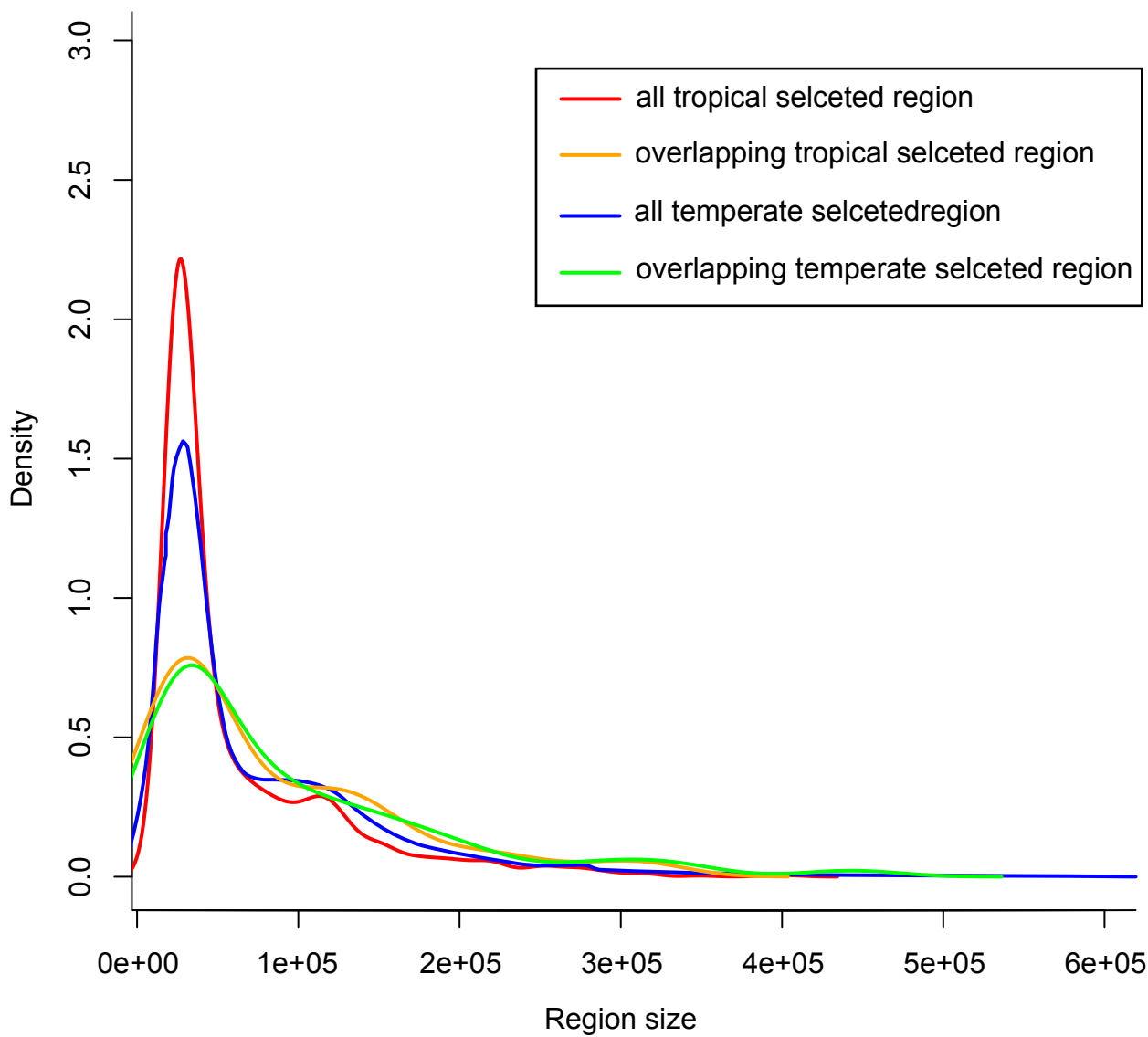

Supplement: S5 Fig — (PDF) [file pone.0169806.s005.pdf]

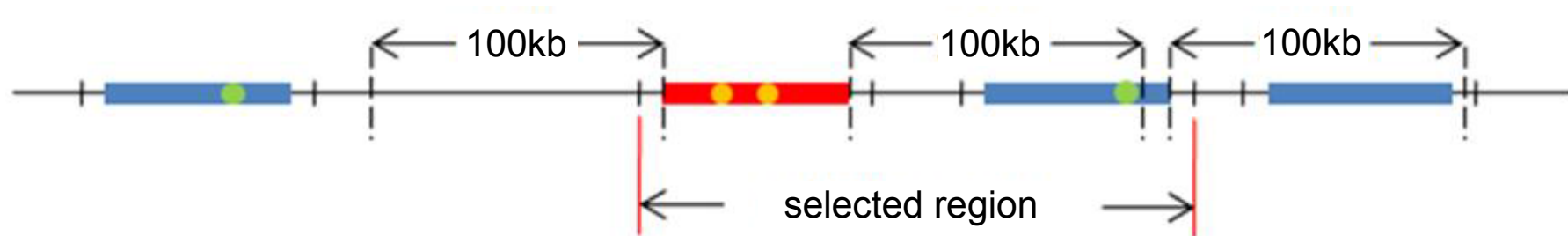

- 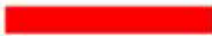 anchoring gene
- 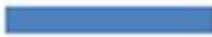 other gene
- 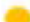 top 5% XP-EHH score SNP
- 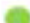 top 10% XP-EHH score SNP

Supplement: S6 Fig — (PDF) [file pone.0169806.s006.pdf]
