# Supplementary material for: A Gene-Oriented Haplotype Comparison Reveals Recently Selected Genomic Regions in Temperate and Tropical Maize Germplasm
Source: PLoS One. 2017 Jan 18;12(1):e0169806. doi: 10.1371/journal.pone.0169806 (PMC5242465; doi:10.1371/journal.pone.0169806)
Supplement: S4 Table — aThe number of functional genes in the whole maize genome. bP-value of the hypergeometric-test. (DOC) [file pone.0169806.s010.doc]

S4 Table. Significant enrichment of candidate selected genes in transcription factor families and ubiquitin pathways.

| Class | Background^a^ | Selected region | | | | | | |
| --- | --- | --- | --- | --- | --- | --- | --- | --- |
|  |  |  | Temperate | *P*-value^b^ |  | Tropical | *P*-value^b^ |  |
| bZIP | 108 |  | 6 | 1.14E-02 |  | 2 | 2.11E-01 |  |
| C3H | 89 |  | 1 | 3.18E-01 |  | 5 | 2.31E-03 |  |
| Ringfinger | 557 |  | 17 | 1.51E-02 |  | 9 | 6.70E-02 |  |

^a^The number of all the function genes in the whole maize genome.

^b^*P*-value of the hypergeometric test.
